# Potential New Applications of Sodium–Glucose Cotransporter-2 Inhibitors Across the Continuum of Cancer-Related Cardiovascular Toxicity

**DOI:** 10.3390/ph18060857

**Published:** 2025-06-09

**Authors:** Agnieszka Maria Zebrowska, Anna Borowiec

**Affiliations:** 1 Cardinal Stefan Wyszynski Institute of Cardiology, 04-628 Warsaw, Poland; agnieszka.zebrowska2@nio.gov.pl; 2 Non-Commercial Clinical Trials Outpatient Clinic, Maria Sklodowska-Curie National Research Institute of Oncology, 02-781 Warsaw, Poland

**Keywords:** sodium–glucose cotransporter-2 inhibitors, cardiotoxicity, cancer therapy-related cardiac dysfunction, cancer therapy-related cardiovascular toxicity, anthracycline-induced cardiotoxicity, cardioprotection, anthracyclines, cardio-oncology

## Abstract

Sodium–glucose cotransporter-2 inhibitors (SGLT2i), initially developed for the management of type 2 diabetes mellitus, have demonstrated significant nephroprotective and cardioprotective effects. These benefits have led to their inclusion in heart failure (HF) management guidelines, irrespective of glycemic status and left ventricular ejection fraction (LVEF). Various anticancer therapies, particularly anthracyclines, are associated with substantial cardiotoxicity risks, resulting in cancer therapy-related cardiovascular toxicity (CTR-CVT). Promising evidence from preclinical and observational studies indicates that SGLT2i may mitigate cardiotoxic effects of cancer therapy by alleviating LVEF decline, reducing HF incidence and hospitalizations, and lowering overall mortality. Moreover, improved survival has been reported in patients with various malignancies. The current review explores the potential applications of SGLT2i in the prevention of CTR-CVT, highlights their possible mechanisms of cardioprotection, discusses the published evidence, and emphasizes the need for the results from ongoing randomized controlled trials to establish SGLT2i efficacy and safety in cardio-oncology patients.

## 1. Introduction

The use of effective anticancer therapies, such as anthracyclines, has significantly improved cancer survival rates [1]. However, these treatments are sometimes associated with cardiotoxicity, which can manifest as a spectrum ranging from asymptomatic mild left ventricular dysfunction to severe symptomatic congestive heart failure (HF) with a reduced left ventricular ejection fraction (LVEF). Moreover, advanced cancer-targeted therapies, often used concurrently, may contribute to other forms of cardiovascular toxicity like cardiac arrhythmias, hypertension, vascular toxicities, and myocarditis, which can limit their use [2,3]. With the growing population of cancer survivors, there is an urgent need to address the cardiovascular complications associated with oncological treatments [4,5]. Sodium–glucose cotransporter-2 inhibitors (SGLT2i), originally used for glycemic control in type 2 diabetes mellitus (DM2), have shown pleiotropic effects, including nephroprotection and cardioprotection [6]. The beneficial effects of SGLT2i were shown in randomized controlled trials [7,8,9,10,11]. In the EMPA REG OUTCOME trial, empagliflozin reduced cardiovascular and overall mortality, HF rate, and hospitalizations, improving clinical outcomes in patients with DM2, established cardiovascular disease, and chronic kidney disease [12,13]. In the AMI-PROTECT Registry, diabetic patients with acute myocardial infarction using SGLT2i had lower hospital mortality, arrhythmic burden, and incidence of contrast-induced acute kidney injury [14]. In particular, the 2022 AHA/ACC/HFSA guidelines for the management of HF highlighted that SGLT2i is a unique class of treatment that plays a role in the full spectrum of HF management, not only in treatment but also prevention [15]. Due to evidence from RCTs, SGLT2i is currently recommended for heart failure patients regardless of LVEF and DM2 status to reduce the risk of HF hospitalizations and cardiovascular (CV) death [8,10,16]. Furthermore, SGLT2i are recommended for the prevention of HF in patients with diabetic chronic kidney disease [6,16,17]. Duan et al. reviewed the antiarrhythmic potential of SGLT2i, suggesting a reduced incidence of new-onset atrial fibrillation and a lower incidence of ventricular arrhythmias in patients with DM2 based on published results of randomized controlled trials (RCTs) [18]. In contrast, a recent meta-analysis including 38 randomized controlled trials (excluding patients with cancer) demonstrated no significant reduction in the incidence of ventricular arrhythmias among SGLT2 inhibitor users. However, the analysis confirmed a beneficial effect of SGLT2 inhibitors in reducing the burden of atrial arrhythmias [19]. Unfortunately, data on patients with active oncological disease were not analyzed in SGLT2i RCTs, and that information was not provided, even in supplementary materials [7,8]. Therefore, in light of the evidence from observational and animal studies, there is a need to evaluate the efficacy and safety of SGLT2i in oncological patients in prospective clinical trials. Therapy with beta-blockers, angiotensin-converting enzyme inhibitors/angiotensin receptor blockers, and mineralocorticoid receptor antagonists has been shown to effectively mitigate the decrease in the left ventricular ejection fraction in patients during oncological therapy [20]. Accordingly, neurohormonal blockade and statins are recommended in the primary prevention of cancer therapy-related cardiovascular toxicity (CTR-CVT) in patients with high and very high risk [4]. There are certain contraindications to the use of neurohormonal blockade (e.g., hypotension) and statins (elevated transaminases), which can limit the use of these therapies. However, SGLT-2 inhibitors are safe and free from these adverse effects, making them a promising and effective preventive strategy. This review examines the emerging role of SGLT2i in preventing CTR-CVT, emphasizing the need to integrate these agents into cardio-oncology practices. It explores the mechanisms underlying their cardioprotective effects, summarizes clinical evidence, and identifies research gaps and future directions.

## 2. Methodology

The current review was conducted by searching multiple databases, including PubMed, Scopus, Web of Science, and Google Scholar, for relevant and up-to-date articles and ongoing clinical trials regarding the use of SGLT2i in the prevention of cardiotoxicity during cancer treatment. Additional sources were identified through citation tracking of original papers and reviews. Papers published before 25 March 2025 were analyzed.

## 3. Cardiotoxicity—Definitions

The definition of cardiovascular toxicities of cancer therapies was proposed in the 2022 consensus statement of the International Cardio-Oncology Society (IC-OS) [6] and integrated into the recent 2022 European Society of Cardiology (ESC) Guidelines on cardio-oncology [4].

### 3.1. Cancer Therapy-Related Cardiovascular Toxicity (CTR-CVT)

The term cancer therapy-related cardiovascular toxicity encompasses various forms of myocardial damage and cardiovascular complications that arise as a result of cancer treatments. It can be the result of various cancer therapies, including chemotherapy (especially anthracyclines), radiation, or targeted therapies. CTR-CVT covers cardiomyopathy, HF, myocarditis, vascular toxicities, hypertension, arrhythmias, corrected QT interval prolongation, and pericardial and valvular heart diseases [4].

### 3.2. Cancer Therapy-Related Cardiac Dysfunction (CTRCD)

CTRCD is used as a standardized term to refer to the wide range of cardiac dysfunctions, including cardiomyopathy and heart failure. CTRCD specifically refers to the adverse effects of cancer treatments on cardiac structure and function. It may be asymptomatic or present clinically as symptomatic HF of varying severity, ranging from mild to severe. In its very severe form, CTRCD manifests as HF requiring inotropic support, mechanical circulatory support, or consideration of heart transplantation. On the other hand, mild asymptomatic CTRCD is defined as preserved LVEF (≥50%) with a new relative decline in GLS by ≥15% from baseline and/or a new increase in cardiac biomarkers [4]. Additionally, CTRCD can lead to long-term or delayed cardiac complications, necessitating lifelong surveillance [2].

### 3.3. Current Methods of Prophylaxis for CTRCD

Neurohormonal therapies administered during anthracycline chemotherapy have been shown in small RCTs to reduce the risk of significant LVEF decline. Meta-analyses support the use of RAAS blockers, beta-blockers, and mineralocorticoid receptor antagonists to prevent LVEF reduction, although they have not significantly impacted overt heart failure or clinical outcomes. These agents also address other cardiac complications of cancer therapy, such as hypertension, arrhythmias, and heart failure. According to the 2022 ESC Cardio-Oncology Guidelines, RAAS blockers, beta-blockers, mineralocorticoid receptor antagonists, and statins should be considered in patients at high or very high risk of cardiotoxicity as they may reduce LVEF decline and myocardial injury [4].

## 4. Oncological Therapies

### 4.1. Anthracycline-Induced Cardiotoxicity (AIC)

Anthracyclines, such as doxorubicin, daunorubicin, and epirubicin, are key agents in the treatment of solid and hematologic malignancies, including breast cancer, lymphoma, and sarcoma. However, among various cancer therapies, anthracyclines are uniquely characterized by their intrinsic potential to induce cardiotoxicity through well-defined mechanism-based pathways [21,22]. They are widely recognized as the primary contributors to cancer therapy-related cardiac dysfunction, primarily driven by myocardial cell damage. This damage leads to myocardial injury, which can clinically manifest itself as heart failure and arrhythmias [3]. Anthracycline-induced cardiotoxicity can present in acute or chronic form. The acute form develops within days after initiating therapy and often manifests as reversible myopericarditis or arrhythmias. The chronic form typically occurs within the first year after anthracycline treatment but can also emerge years later, resulting in heart failure with a reduced LVEF. Recent data suggest that acute and chronic cardiotoxicity represent different stages of the same pathological spectrum [22]. AIC is dose-dependent and may ultimately result in an irreversible decline in cardiac function. Age (being very young or old) is an important risk factor associated with CTRCD; other important factors include a history of cardiovascular disease, including heart failure, coronary artery disease, hypertension, diabetes, chronic kidney disease, obesity, smoking, and elevated baseline cardiac biomarkers [23]. Radiation therapy and other cardiotoxic therapies administered simultaneously with anthracyclines increase the risk of both early and late cancer therapy-related cardiovascular toxicity [4]. Notably, anthracycline-induced cardiotoxicity may develop years or even decades after therapy, representing a significant long-term risk for cancer survivors. Data on its prevalence remain limited and inconsistent, largely due to evolving definitions of cardiotoxicity, variability in diagnostic methods, and the wide temporal range of AIC manifestation, which spans from days to more than 20 years after treatment. The incidence of CTRCD reported in the literature ranges from less than 2% at a cumulative dose of doxorubicin of 300 mg/m^2^ to 3–5% at 400 mg/m^2^ and increases substantially to 18–48%, with cumulative doses reaching 700 mg/m^2^ [3,22,24]. Data from a Brazilian registry demonstrated a high incidence of cancer therapy-related cardiovascular toxicity in children and adolescents with cancer. Vascular complications were reported in 141 patients (43.3%), with systemic arterial hypertension being the most frequent. Furthermore, CTRCD following anthracycline treatment was observed in 84 (25.8%) patients [25]. Differences in patient characteristics, including age, cancer type, treatment regimen, concomitant therapies, emerging treatments, cardiological assessment methods, and time of surveillance, make it challenging to identify consistent cardiotoxicity patterns. 

#### Molecular Mechanisms of Anthracycline-Induced Cardiotoxicity

The detrimental impact of anthracyclines on the cardiovascular system is a multifactorial process. The proposed pathways can be broadly classified into mechanisms based on oxidative stress, mitochondrial dysfunction, inflammation, and cell death [26]. Anthracyclines disrupt mitochondrial respiratory chain complexes, leading to reactive ROS generation and increased calcium concentrations that later cause mitochondrial and DNA damage, which subsequently promotes cardiomyocyte apoptosis [27]. Further DNA damage from topoisomerase inhibition results in cell death [28]. AIC is also associated with a decreased expression of AMPK (5’ AMP-activated protein kinase), which, along with the induction of double-strand breaks and apoptosis, is the result of DNA intercalation and inhibition of topoisomerase II (TOP2) enzymes, and this plays a key role in the cardiotoxic process [29]. Cardiomyocytes are highly vulnerable to oxidative stress due to their limited regenerative capacity. This leads to impaired contractility, calcium dysregulation, fibrosis, and finally heart failure [30,31]. Oxidative stress further damages cell membranes and DNA, promoting cardiomyocyte apoptosis and disrupting calcium homeostasis, thus contributing to a reduced ejection fraction and increased risk of arrhythmia [26,31,32].

### 4.2. Thoracic Radiation Therapy

Approximately half of all cancer patients undergo radiation therapy (RT) for curative or palliative purposes [1]. Patients with breast, lung, and esophageal cancer, as well as those with mediastinal malignancies such as lymphoma, are at high risk of developing radiation-induced heart disease (RIHD), which can manifest as coronary artery disease, heart failure, and valvular heart disease [4,33]. The risk of early and late cancer therapy-related cardiovascular toxicity is further increased when RT is administered concurrently with chemotherapy, particularly anthracyclines [4]. The primary known mechanism underlying RT-induced cardiac damage involves collagen deposition and fibrosis [34]. Given the antifibrotic properties demonstrated by SGLT2 inhibitors in preclinical studies, these drugs present a promising but yet-to-be-established therapeutic approach for mitigating RIHD [35].

### 4.3. Targeted Therapies

Human epidermal growth factor receptor 2 (HER-2) is a transmembrane tyrosine kinase receptor that is overexpressed in several types of malignancies, including breast, ovarian, gastrointestinal, and bladder cancers. Notably, HER-2 expression has also been detected in adult cardiomyocytes, suggesting its role beyond oncological processes. Trastuzumab, a humanized monoclonal antibody directed against HER-2, is a key component of targeted therapy in HER-2-positive malignancies. However, its administration has been associated with cardiotoxicity, primarily manifested by impaired cardiomyocyte contractile function and disturbed calcium homeostasis. These functional alterations occur in the absence of direct cell death and are thought to result largely from mitochondrial dysfunction and disruptions in cellular energy metabolism [36]. The results of a study by Gordon L.I. et al. suggest that erbB2 plays a role in cardiomyocyte survival and that the deleterious effects of trastuzumab on the heart occur through a mitochondrial pathway and are mediated by ROS production [37]. The incidence of cardiac dysfunction associated with trastuzumab therapy has been reported to be approximately 6% [38]. The risk factors for cancer therapy-related cardiac dysfunction in patients receiving HER-2 targeted therapy overlap with traditional cardiovascular risk factors, such as advanced age and elevated body mass index. Additionally, other factors have been identified, including race, a lower baseline left ventricular ejection fraction, elevated systolic blood pressure, the presence of coronary artery disease, diabetes, and arrhythmias, and prior exposure to anthracycline-based chemotherapy [39]. Trastuzumab-related CTRCD typically presents as left ventricular systolic dysfunction or symptomatic heart failure. Notably, in contrast to anthracycline-induced cardiotoxicity, trastuzumab-related cardiotoxic effects are generally reversible and not directly dose-dependent. However, the risk of developing trastuzumab-induced cardiotoxicity is significantly increased in patients with prior exposure to anthracyclines, suggesting a synergistic cardiotoxic effect [40]. Preclinical studies have shown that SGLT2 inhibitors may attenuate trastuzumab-induced cardiotoxicity by limiting DNA damage as well as by restoring intracellular calcium homeostasis. These protective mechanisms translated into an improved left ventricular ejection fraction in animal models [41].

### 4.4. Immunotherapy

Immune checkpoint inhibitors (ICIs), which have proven effective in treating a variety of malignancies, are increasingly being used in oncology. However, their use has been associated with immune-related cardiotoxicity. Although cardiovascular complications related to ICIs are infrequent, occurring in less than 1% of patients, they can be life-threatening [42]. Among the immune-related cardiovascular adverse effects, myocarditis is the most prevalent and most severe. Less common manifestations of ICI-related cardiotoxicity include pericardial disease, arrhythmias, Takotsubo-like syndrome, and acute myocardial infarction, all of which remain insufficiently characterized [41]. ICIs target immune checkpoints such as Programmed Cell Death Protein 1 (PD-1) and its ligand (PD-L1), as well as Cytotoxic T-Lymphocyte Antigen 4 (CTLA-4), leading to an enhanced immune response against tumors. However, this heightened inflammatory response may also affect cardiac tissue and arterial structures [43]. Emerging evidence suggests a link between ICI therapy and accelerated atherosclerosis, as well as an increased risk of atherosclerotic cardiovascular events, particularly myocardial infarction [43]. To explore this issue, Perelman et al. [44] conducted a retrospective analysis investigating the potential cardioprotective role of SGLT2 inhibitors in patients treated with immune checkpoint inhibitors, with a particular emphasis on CTR-CVT. Their findings indicated that pretreatment with SGLT2i was associated with reduced all-cause mortality in patients receiving ICI therapy. Importantly, no cases of myocarditis or atrial fibrillation were reported in the SGLT2i-treated cohort; nevertheless, the small sample size (n = 28) precludes definitive conclusions. The authors further proposed that the cardioprotective effects of SGLT2 inhibitors may be mediated through the suppression of systemic inflammation and myocardial injury, potentially via the modulation of immune and inflammatory responses.

### 4.5. Hormone Therapy

Gonadotropin-releasing hormone agonists (GnRH-As) are widely used in hormone-sensitive cancers such as breast and prostate cancer [45]. However, they are associated with an increased risk of adverse CV events, including coronary artery disease, HF, and stroke [46]. In patients with prostate cancer undergoing hormone therapy, the use of SGLT2 inhibitors was associated with significantly reduced all-cause mortality as well as lower risks of heart failure, HF exacerbations, peripheral artery disease, atrial fibrillation, cardiac arrest, and the need for renal replacement therapy. Moreover, SGLT2i use was linked to fewer emergency department visits and hospitalizations in this population [47]. Similarly, a retrospective study evaluating patients with prostate cancer receiving hormone therapy with GnRH-As demonstrated a favorable effect of SGLT2 inhibitors in individuals with type 2 diabetes. Treatment with SGLT2 inhibitors has been associated with a lower risk of all-cause mortality and reduced incidence of heart failure and myocardial infarction when compared to non-SGLT2i users [48].

## 5. Multifaceted Cardioprotective Mechanisms of SGLT2i

**Figure 1:**
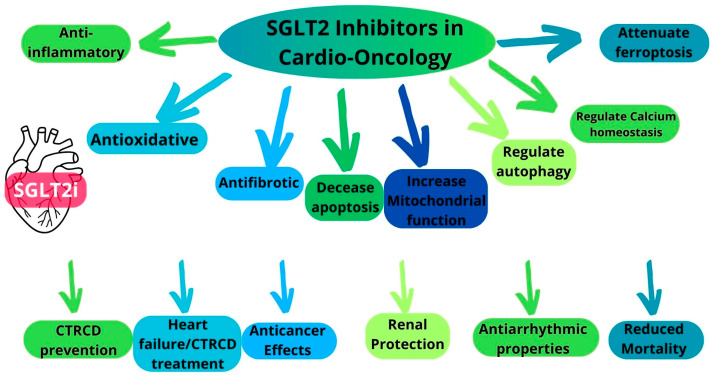
SGLT2 inhibitors in cardio-oncology.

Preclinical studies suggest that SGLT2 inhibitors exert a broad spectrum of cardioprotective effects that may prevent CTR-CVT, Figure 1. Their protective actions include a reduction in inflammation, oxidative stress, apoptosis, and fibrosis, alongside an enhancement of cardiomyocyte viability, regulation of autophagy, prevention of degenerative changes, and attenuation of cell death. Furthermore, SGLT2i not only mitigate structural and functional cardiac damage induced by anticancer therapies but may also improve the efficacy of chemotherapy by limiting its cardiotoxic side effects. SGLT2 inhibitors exhibit a broad range of cardioprotective effects that may counteract CTRCD. Studies have demonstrated that SGLT2 inhibitors attenuate inflammation [27,28,49,50,51], prevent cell death [27,49,52,53], and reduce oxidative stress by inhibiting the generation of ROS [27,53,54] and modulating other pathways [50,55,56,57]. Additionally, SGLT2 inhibitors help restore ion homeostasis and enhance intracellular calcium handling. This prevents a calcium overload, a key contributor to mitochondrial dysfunction in cardiotoxicity [58]. Furthermore, SGLT2 inhibitors enhance mitochondrial biogenesis, dynamics, and metabolic efficiency [59]. Collectively, these mechanisms contribute to enhanced myocardial energy metabolism and increased cardiomyocyte survival [27]. Moreover, SGLT2 inhibitors promote a shift toward ketone body metabolism, further reducing oxidative stress and improving myocardial energy efficiency [58].

## 6. Overview of the Preclinical Evidence of the Cardioprotective Effects of SGLT2i

Several studies have highlighted the beneficial effects of SGLT2 inhibitors in cellular and animal models of cardiotoxicity. SGLT2 inhibitors in cardio-oncology In non-diabetic mice, SGLT2 inhibitors facilitated the restoration of cell viability and calcium homeostasis and reduced inflammation. They also attenuated ferroptosis, decreased the expression of xanthine oxidase, and reduced reactive oxygen species (ROS) production, resulting in a reduction in oxidative stress [27]. SGLT2 inhibitors help restore calcium homeostasis through the Na^+^/H^+^ exchanger, leading to a reduced concentration of intracellular Ca^2^⁺ in cardiomyocytes. This mechanism may contribute to their potential antiarrhythmic effect and beneficial impact on cardiomyocyte contractility [27].

In an animal model, SGLT2 inhibitors activated the PI3K/Akt signaling pathway following anthracycline treatment, enhancing antioxidant defenses (HO-1, NQO1, and SOD) and improving mitochondrial function via Nrf2. This reduction in oxidative stress was associated with decreased hypertrophy, as evidenced by lower concentrations of ANP and BNP and reduced fibrosis, reflected by a lower expression of phospho-Smad3, collagen I, fibronectin, and α-SMA. Additionally, IL-8 levels decreased, likely through the PI3K/Akt/Nrf2/p38/NF-κB signaling cascade, indicating an anti-inflammatory effect of SGLT2 inhibitors. In vivo validation demonstrated improved cardiac function on echocardiography in SGLT2i-treated rats, supporting their potential to counteract anthracycline-induced cardiotoxicity by targeting oxidative stress, fibrosis, hypertrophy, and inflammation [49].

In addition to their antioxidant properties, the protective effects of SGLT2 inhibitors on cardiomyocytes following anthracycline exposure may be attributed to their ability to mitigate lipid peroxidation [29]. SGLT2 inhibitors also exhibit anti-inflammatory effects in cardiomyocytes by downregulating the NLRP3 and MyD88 pathways, leading to reduced activation of NF-κB and lower levels of pro-inflammatory cytokines, such as IL-1β, IL-6, and IL-8 [28].

Barış et al. demonstrated in an animal model that empagliflozin significantly ameliorated doxorubicin-induced acute cardiotoxicity by inhibiting DNA double-strand breaks [60]. Additionally, the concurrent use of SGLT2 inhibitors in an anthracycline-pretreated animal model reduced the left ventricular size and infiltrative cell proliferation while promoting normal cell morphology.

In an animal model of chronic kidney disease and anthracycline-induced cardiomyopathy, SGLT2 inhibitors were found to reduce oxidative stress by inhibiting NADPH oxidase 1 and 2 as well as the PI3K/Akt signaling pathway while lowering levels of oxidized proteins. As a result, SGLT2 inhibitors protected against LVEF decline and adverse cardiac remodeling [56]. SGLT2 inhibitors have demonstrated the ability to suppress oxidative stress and improve energy metabolism in an animal model treated with anthracyclines, specifically by activating the AMPK/SIRT1/PGC-1α pathway [57]. SGLT2 inhibitors enhanced AMPK and SIRT1 activation, leading to a reduction in oxidative stress, normalization of mitochondrial structure and function, suppression of inflammation, minimization of coronary microvascular injury, improved contractile performance, and attenuation of cardiomyopathy development, as demonstrated in animal models [57].

In vivo, pretreatment with SGLT2 inhibitors decreased the phosphorylation of the JNK (c-Jun N-terminal kinase)/STAT3 axis as well as the generation of ROS and oxidized nicotinamide adenine dinucleotide (NAD^+^), thereby inhibiting apoptosis. Clinically, this resulted in reduced fibrosis and improved cardiac function [55].

A study using mouse cardiomyocytes pretreated with SGLT2 inhibitors prior to anthracycline exposure demonstrated that SGLT2 inhibitors attenuated mitochondrial ROS production, inhibited the oxidation of Ca^2+^/calmodulin-dependent protein kinase II (ox-CaMKII), and reduced CaMKII-dependent phosphorylation of the ryanodine receptor 2 (RyR2). These effects improved calcium handling, increased the expression of sodium-calcium exchanger 1 (NCX1), and contributed to the restoration of ion homeostasis, ultimately leading to reduced cardiac dysfunction compared to myocytes without SGLT2 inhibitor pretreatment [61]. Recent findings from a preclinical trial using a translatable large animal model (pigs) demonstrated that SGLT2 inhibitors prevent anthracycline-induced systolic dysfunction in a dose-dependent manner. The protective effect of 20 mg empagliflozin was associated with improvements in LVEF, increased utilization of ketone bodies, enhanced myocardial energy metabolism, and the preservation of mitochondrial integrity and function [62].

Interestingly, in preclinical models of anthracycline-induced cardiotoxicity, SGLT2 inhibitor pretreatment significantly reduced heart failure biomarkers, specifically troponins and NT-pro-BNP, while improving cardiac function, thereby demonstrating its cardioprotective properties [27,28,49,50,52,54,55,56,57,58,60,61].

### Antifibrotic Properties of SGLT2i

The antifibrotic properties of SGLT2 inhibitors, including a reduction in collagen expression, depletion of collagen deposition, and inhibition of profibrotic pathways such as TGF-β/SMAD signaling, as well as pathways potentially mediated by angiotensin II, play a crucial role in attenuating adverse cardiac fibrosis and remodeling [27,56,63]. In a murine model, SGLT2 inhibitors were shown to attenuate myocardial fibrosis, primarily through the inhibition of the transforming growth factor β (TGF-β)/Smad signaling pathway, alongside the activation of the nuclear factor erythroid 2–related factor 2 (Nrf2)/antioxidant response element pathway, thus contributing to their antifibrotic and cardioprotective effects [29]. Notably, a study by Sabatino et al. demonstrated that empagliflozin pretreatment in mice receiving anthracycline therapy effectively preserved the LVEF and reduced myocardial fibrosis, independently of the presence of diabetes [58]. Similarly, reduced fibrosis after anthracycline exposure was observed in a large animal model. Although the difference in MRI-assessed fibrosis did not reach statistical significance—potentially due to the limited sample size—histological evaluation confirmed a lower extent of myocardial fibrosis in animals treated with 20 mg of empagliflozin, as reported by Medina-Hernández et al. [62].

It is important to note that in all the presented preclinical models of anthracycline-induced cardiotoxicity, doxorubicin was used. Among the SGLT2 inhibitors, only dapagliflozin [28,49,50,52] and empagliflozin [27,54,55,56,57,58,60,61] were utilized in the studies mentioned above.

## 7. Clinical Evidence for the Cardioprotective Properties of SGLT2i

### 7.1. Retrospective Studies

Several retrospective studies have reported favorable clinical outcomes in patients receiving SGLT2 inhibitors concomitantly with cancer therapy.

Gongora et al. reported a significant reduction in all-cause mortality and cardiovascular events, including heart failure incidence, heart failure-related hospitalizations, new-onset cardiomyopathy, and clinically significant arrhythmias, in diabetic patients treated with SGLT2 inhibitors during anthracycline-based chemotherapy for hematologic malignancies [64]. A further study by Abdel-Qadir et al. evaluated an older population (aged >65 years) with type 2 diabetes mellitus and no prior history of heart failure [65]. The study demonstrated that patients receiving anthracycline-based chemotherapy who were concurrently treated with SGLT2 inhibitors exhibited significantly lower rates of heart failure hospitalizations compared to those not receiving SGLT2i therapy.

Chiang et al. investigated the cardioprotective effects of SGLT2 inhibitors in a cohort of diabetic breast cancer patients undergoing anthracycline-based chemotherapy [66]. In that study, SGLT2i use was associated with a significantly lower risk of heart failure hospitalizations and improved overall survival compared to non-users. However, it is noteworthy that only 8% of the patients in this cohort received anthracyclines, which may limit the generalizability of the findings specifically to anthracycline-induced cardiotoxicity.

In another study by Hwang et al., conducted in an Asian population, the analysis of cancer patients demonstrated a 20% reduction in cardiovascular events, including heart failure hospitalization, acute myocardial infarction, ischemic stroke, and all-cause mortality in patients receiving SGLT2 inhibitors compared to both non-diabetic individuals and diabetic patients not treated with SGLT2i [67].

A unique study by Avula et al. assessed the efficacy of SGLT2i use in managing CTRCD resulting from various anticancer therapies in diabetic patients [68]. Among 640 matched patients receiving guideline-directed medical therapy (GDMT) with or without SGLT2i, those using SGLT2i demonstrated significant reductions in acute heart failure exacerbation, atrial fibrillation or flutter, all-cause hospitalizations or emergency department visits, all-cause mortality, and renal complications, namely acute kidney injury and the need for renal replacement therapy.

With a larger study population, Bhatti et al. [69], from the same investigative team as Avula et al., conducted a retrospective analysis with 8675 diabetic patients with cancer treated with SGLT2 inhibitors. Their findings demonstrated significantly lower rates of CTRCD in SGLT2i users compared to non-users. Moreover, the SGLT2i group exhibited a reduced incidence of heart failure, all-cause mortality, and all-cause hospitalizations. Notably, the occurrence of new-onset atrial fibrillation or flutter was also lower among patients receiving SGLT2i. Unfortunately, the data regarding the safety profile of SGLT2i in this population were not provided.

A large observational study involving 1412 propensity score-matched patients undergoing anthracycline therapy demonstrated that SGLT2 inhibitor use was associated with a significant reduction in the incidence of new-onset heart failure and arrhythmias over a two-year follow-up period. However, no significant differences were observed between the SGLT2i and control groups regarding all-cause mortality, myocardial infarction, or all-cause hospitalizations. Importantly, the authors emphasized the favorable safety profile of SGLT2 inhibitors, which was comparable to the placebo [70].

A synthesized summary of available retrospective studies and one prospective study indicates that the use of SGLT2 inhibitors in oncology patients, particularly those with type 2 diabetes, is associated with a significant reduction in all-cause mortality and heart failure hospitalizations. Among the 15 retrospective studies, 13 (86.7%) reported a reduction in overall mortality, and 9 out of 10 studies reporting HF hospitalization data showed a reduction in HF-related admissions. Additional benefits included lower rates of new-onset or worsening HF, arrhythmias, acute kidney injury, the need for renal replacement therapy, and major adverse cardiovascular events such as myocardial infarction and ischemic stroke. One prospective case–control study (EMPACARD) did not demonstrate a difference in mortality or HF hospitalization; however, it reported favorable cardiac imaging outcomes, including a smaller decline in the LVEF and lower impairment in global longitudinal strain. The protective effect of SGLT2i was observed across various cancer types, including breast, gastrointestinal, prostate, lymphoma, and others, and in patients undergoing diverse anticancer therapies ranging from anthracyclines to immune checkpoint inhibitors and hormonal treatments. Despite the variability in cancer types, treatment regimens, and patient populations, the consistency of findings across studies suggests a potential class effect of SGLT2 inhibitors in mitigating cardiovascular toxicity in oncology settings.

It is important to acknowledge that the majority of the included studies are retrospective and observational in nature, which introduces a potential risk of bias. The wide variation in cancer types, treatment modalities, and SGLT2i use also limits the ability to draw definitive conclusions. Nonetheless, the consistency of the observed trends across different cohorts supports further investigation in prospective randomized settings.

### 7.2. Case Reports

Currently, there are no case studies specifically examining the use of SGLT2 inhibitors to prevent cardiotoxicity in oncological patients. Although guidelines recommend SGLT2 inhibitors for patients with heart failure, few case reports involve cancer patients. In one such report, seven patients with anthracycline-induced cardiac dysfunction demonstrated significant improvements in the New York Heart Association (NYHA) functional class and LVEF after 24 weeks of SGLT2i therapy [71]. In another case, a cancer survivor with anthracycline-induced cardiomyopathy experienced an improvement in the LVEF from 18% to 52% over two years while on optimal medical therapy, which included empagliflozin [72].

### 7.3. Prospective Studies

To date, only one prospective case–control study has assessed the cardioprotective effects of empagliflozin in high-risk breast cancer patients undergoing anthracycline therapy. Among the 76 patients enrolled, those treated with empagliflozin (10 mg/day) demonstrated a significant reduction in the incidence of CTRCD compared to the control group. Additionally, empagliflozin preserved the LVEF and global longitudinal strain (GLS). However, no significant differences were observed in secondary outcomes, including mortality or hospitalization due to heart failure [73].

Currently, at least five ongoing and four planned RCTs are investigating the cardioprotective effects of SGLT2 inhibitors.

One such actively recruiting, randomized, and double-blind clinical trial, namely the EMPACT study (Empagliflozin in the Prevention of Cardiotoxicity in Cancer Patients Undergoing Chemotherapy Based on Anthracyclines; NCT05271162), aims to evaluate the efficacy and safety of empagliflozin (10 mg) administered prior to anthracycline-based chemotherapy to prevent CTRCD in patients without pre-existing heart failure. This prospective study will assess the LVEF and global longitudinal strain (GLS) over a 12-month follow-up period. Secondary outcomes include the LVEF as measured by cardiac magnetic resonance imaging, biomarkers, and the incidence of cardiovascular complications.

### 7.4. Discussion

Sodium–glucose cotransporter-2 inhibitors have emerged as a transformative class of anti-diabetic agents, providing significant cardiovascular and renal benefits beyond glycemic control. Recent studies have expanded their potential applications in oncology, particularly in mitigating CTR-CVT and improving overall outcomes for cancer patients. The promising role of SGLT2i in preventing CTR-CVT marks an important advancement in the field of cardio-oncology. The existing evidence underscores the cardioprotective benefits of SGLT2i in counteracting the detrimental effects of anthracyclines [64,65,66,67,68,69,70,73,74], immune checkpoint inhibitors [44], and hormonal therapy [47,48]. The mechanisms underlying these benefits are multifaceted, including a reduction in inflammation, improvement in mitochondrial function, and enhancement of metabolic efficiency, all of which contribute to cardiovascular protection.

The case reports, registry-based studies, retrospective cohort studies, and preclinical research presented above constitute the currently available body of evidence regarding the cardioprotective role of SGLT2i in oncological treatment. While retrospective studies offer valuable initial insights into the potential benefits of SGLT2i during cancer treatment, prospective clinical trials are essential to definitively assess the therapeutic effectiveness, safety, and long-term outcomes of these agents in the cancer patient population. So far, the EMPACARD-PILOT trial by Daniele et al. stands as the first and only published randomized study providing evidence for empagliflozin’s potential in mitigating anthracycline-induced cardiac dysfunction. However, the small sample size and the absence of blinding limit the strength of its conclusions. Despite these limitations, the observed preservation of the LVEF and global longitudinal strain suggests a meaningful cardioprotective effect of SGLT2 inhibitor treatment [73].

Several meta-analyses have explored the cardioprotective effects of SGLT2 inhibitors. Notably, the meta-analysis by Tabowei was limited by a small number of available retrospective studies, with only one-fourth of participants receiving SGLT2 inhibitors. Furthermore, these four observational studies used different SGLT2 inhibitors and varied substantially in the follow-up duration, which could have influenced key outcomes, such as mortality or the onset of heart failure. The lack of detailed mortality data precludes definitive conclusions. Although these findings cannot be directly extrapolated, they provide valuable insights from high-risk patient groups that could inform the development of cardioprotective strategies [75].

However, it is important to acknowledge the diversity among the analyzed observational studies, particularly the heterogeneity in the type, dosage, and timing of SGLT2i administration relative to cancer treatment. Moreover, the patient populations in these studies varied in terms of cancer types (not equally represented), disease stages, and oncological treatments. The duration of follow-up also differed across studies. Current meta-analyses of studies on SGLT2i use during cancer therapy include patients from various cancer types and treatment regimens. Despite significant heterogeneity, the findings indicate a beneficial effect of SGLT2i, with a lower prevalence of CTRCD and improved overall survival [75,76].

Anthracyclines are among the most extensively studied oncological treatments due to their long-standing use, well-established therapeutic role, and proven causality in cardiotoxicity. Given their recognized cardiotoxic effects, identifying cardioprotective agents has become a key area of research. The majority of available studies in this field focus on patients undergoing anthracycline-based therapy. Notably, a recent meta-analysis by Bhalraam et al. reported a stronger cardioprotective effect of SGLT2 inhibitors in breast cancer patients treated with anthracyclines compared to those undergoing other treatments [76].

Additionally, the protective benefit of SGLT2 inhibitors appeared more pronounced in cohorts with a lower proportion of men. However, it remains unclear whether the type of cancer or female sex plays a significant role in this effect. Further research is needed to determine whether SGLT2 inhibitors are especially advantageous for breast cancer patients. Unfortunately, different cancer types and oncological treatment regimens were not equally represented in the analyzed studies, limiting the generalizability of the findings. Many studies focusing on anthracycline-induced cardiotoxicity primarily involve breast cancer, which may explain the female-dominated cohorts. Moreover, studies often fail to provide specific information on sex differences in outcomes. It remains unclear whether the female sex is more susceptible to the protective effects of SGLT2 inhibitors, potentially influenced by hormonal factors, a question that warrants further investigation. In other cancer types, such as sarcomas, higher doses of anthracyclines are often administered. In oncological patients undergoing cancer therapies, it is challenging to draw direct conclusions, as treatment regimens may change due to disease progression or intolerance to therapy.

Although the existing literature suggests a potential protective role of SGLT2 inhibitors in mitigating cancer therapy-related cardiovascular toxicity, several important limitations must be considered when interpreting the current findings. Most published studies are retrospective and observational, often with relatively small cohorts of patients using SGLT2 inhibitors. Additionally, there is a lack of data on cardio-oncology patients without diabetes, as most research has focused on diabetic populations, following prevailing guidelines. Another limitation is the predominance of studies involving elderly patients with multiple comorbidities, which limits the diversity of age groups included in the research. As indications for SGLT2 inhibitors expand, more data on non-diabetic patients will likely emerge. Moreover, the overrepresentation of breast cancer patients in many studies has led to sex-related disparities in the data. The variability in assessment methods—such as differences in imaging techniques, biomarker selection, and definitions of cardiotoxicity—further complicates the accurate detection of cardiac dysfunction. Despite these limitations, retrospective studies suggest a beneficial influence of SGLT2 inhibitors in patients undergoing chemotherapy. However, the role of SGLT2 inhibitors, whether used alongside conventional cardioprotective therapies (such as ACE inhibitors, ARBs, beta-blockers, and statins) or as a standalone strategy for primary prevention, requires further investigation to establish evidence-based guidelines.

## 8. Anticancer Effects of SGLT2 Inhibitors

The emerging data indicate that SGLT2i may possess potential anticancer effects in addition to their well-established metabolic and cardioprotective functions.

A recent meta-analysis conducted by Xu et al. demonstrated that the use of SGLT2 inhibitors was associated with improved oncological outcomes, including a significant reduction in cancer-related mortality and disease progression [77]. These benefits were particularly notable in malignancies characterized by high glucose uptake and glycolytic activity, such as breast and lung cancers. In a large cohort study, Bhatti et al. demonstrated that patients with various malignancies who were treated with SGLT2 inhibitors exhibited a lower incidence of newly diagnosed metastatic disease and a reduced need for systemic antineoplastic therapy compared to non-users [69].

### Proposed Anticancer Mechanisms

The potential anticancer properties of SGLT2 inhibitors are thought to arise from their ability to reduce glucose availability within the tumor microenvironment, attenuate oxidative stress, and influence immune system activity. Through the modulation of key metabolic pathways, SGLT2 inhibitors may impair cancer cell proliferation and promote apoptosis. Importantly, a recent meta-analysis found no increase in the overall risk of cancer, including bladder and breast cancer, among SGLT2 inhibitor users. However, a possible elevated risk of renal cancer has been suggested, warranting further investigation. The existing literature provides emerging evidence supporting a potential anticancer role for SGLT2 inhibitors across various malignancies. As extensively reviewed by Pandey et al. [78], the most compelling data highlight the anticancer activity of SGLT2 inhibitors in lung and breast adenocarcinomas, hepatocellular carcinoma, and pancreatic cancer. These effects are believed to be linked to the modulation of tumor metabolism and cellular signaling pathways. Recent research indicates that SGLT2 is overexpressed in various types of cancer and may contribute to tumor development, progression, and metastasis. These findings suggest that SGLT2 inhibitors could have broad therapeutic applications in oncology. While SGLT1 primarily facilitates glucose absorption in the small intestine, SGLT2 is chiefly responsible for glucose reabsorption in the kidneys, accounting for over 80% of the filtered glucose reabsorption in the proximal tubule. Notably, beyond renal tissue, SGLT2 expression has also been detected in the mammary glands, testes, liver, lungs, intestines, skeletal muscle, spleen, and cerebellum [79]. SGLT2 inhibitors reduce glucose reabsorption in the renal proximal tubule, resulting in increased urinary glucose excretion and effective glycemic control, independent of insulin. The emerging evidence suggests that SGLT2 is also expressed in various tumor cells. Notably, preclinical studies have demonstrated that dapagliflozin significantly improves survival in murine models of solid tumors, indicating a potential anticancer benefit beyond glycemic control [80]. Additionally, a meta-analysis by Benedetti et al. demonstrated a significant association between SGLT2 inhibitor use and a reduced overall cancer risk compared to the placebo [81]. Finally, in a recent review by Dabour et al. [82], several mechanisms of potential anticancer action have been proposed. These include enhancing anticancer immune response, inhibiting mitochondrial complex I and activating AMPK, inhibiting the phosphoinositide 3-kinase/Akt pathway, and inhibiting glucose uptake.

These findings highlight the potential of SGLT2 inhibitors as a promising therapeutic strategy in oncology. However, while encouraging, these results underscore the urgent need for well-designed clinical trials to directly assess the impact of SGLT2i on cancer incidence, progression, and patient survival.

## 9. SGLT2i Safety

A comprehensive meta-analysis by Wanner et al. evaluated the safety profile of empagliflozin in a pooled population of 10,472 patients from four large placebo-controlled trials, with a median follow-up of 2.1 years [13]. The analysis confirmed a favorable safety profile for empagliflozin, with no increased risk of severe hypoglycemia, bone fractures, or lower limb amputations. The incidence of serious urinary tract infections was similar between the empagliflozin and placebo groups, although a slightly higher rate was observed among female patients. While ketoacidosis and significant volume depletion were rare, they occurred marginally more frequently in the empagliflozin group, with the highest incidence of volume depletion noted in patients aged ≥85 years. Notably, empagliflozin was associated with a reduced risk of acute kidney injury. These findings underscore the established safety of empagliflozin across a wide range of patients, including those with renal impairment, heart failure, and advanced age. However, it is important to note that cancer patients were excluded from these trials, limiting the generalizability of the results to this population and underscoring the need for further research in oncological settings. Another meta-analysis focusing on the Japanese diabetic population demonstrated that the use of SGLT2 inhibitors was associated with comparable risks of hypoglycemia, urinary tract infections, vaginal infections, hypovolemia, and bone fractures when compared to the placebo [83]. Chiang et al. demonstrated the beneficial effects of SGLT2 inhibitors in breast cancer patients treated with anthracyclines, with no increased risk of serious adverse events, such as hypoglycemia or sepsis [66]. Over a 5-year observation period, Henson et al. [74] assessed the safety of SGLT2 inhibitors in patients with a history of anthracycline treatment, focusing on ketoacidosis, yeast infections, and lower extremity amputations. Notably, the incidence of ketoacidosis was higher in the cohort receiving SGLT2 inhibitors. However, this result was based on a small number of events: only 17 patients (1.3%) in the SGLT2i group and 10 patients (0.8%) in the non-SGLT2i group developed ketoacidosis. Additionally, the incidence of yeast infections was comparable between the two groups. A meta-analysis by Agarwal et al. found that the use of SGLT2 inhibitors was associated with significantly fewer overall drug-related adverse events [84]. The most commonly reported adverse effects in observational studies involving oncology patients using SGLT2 inhibitors include urinary tract infections (UTIs), sepsis, and acute kidney injury (AKI). Notably, the frequency of UTIs was lower in patients using SGLT2 inhibitors [64,68], and sepsis rates were also found to be lower in these patients [64,66]. While the results of meta-analyses comparing the rates of AKI remain inconclusive, a recent study by Kuo et al. demonstrated that, among 82,654 cancer patients undergoing chemotherapy with anthracyclines, SGLT2 inhibitor use (n = 19,831) was associated with a lower risk of sepsis and no increased risk of diabetic ketoacidosis [76,85]. In a study by Tang et al. [48], which evaluated the safety and efficacy of SGLT2 inhibitors in diabetic prostate cancer patients undergoing hormone therapy, the use of SGLT2 inhibitors was not associated with an increased risk of urinary tract infections, hypoglycemia, acute kidney injury, or diabetic ketoacidosis. Additionally, no cases of Fournier gangrene were reported in the SGLT2 inhibitor group.

However, given the limited number of observational studies involving oncological patients using SGLT2 inhibitors and the fact that not all of these studies report on complications, caution is warranted.

## 10. All-Cause and Cancer-Specific Mortality with SGLT2 Inhibitors

Published retrospective studies [64,65,67,68,74] and meta-analyses [75,83] consistently demonstrate that the use of SGLT2 inhibitors is associated with a reduction in mortality among diabetic patients undergoing anthracycline-based cancer therapy. In a retrospective analysis by Henson et al., which involved propensity-matched heart failure patients previously treated with anthracyclines, SGLT2i use (n = 1323) was associated with a lower risk of cachexia, malnutrition, adult failure to thrive, and all-cause mortality [74].

These findings are supported by several meta-analyses, which show a clear reduction in overall mortality among patients receiving SGLT2 inhibitors during cancer treatment [75,83,84]. Notably, in a meta-analysis by Bhalraam et al., which included 11 studies, SGLT2i use was associated with a significantly lower risk of mortality, with an impressively low number needed to treat (NNT) of just 4 [76].

Huang et al. reported that the use of SGLT2i in oncology patients was associated with significantly better outcomes for both cancer-specific and all-cause mortality. In their study, the five-year overall survival rate was 92.0%, and the cancer-specific survival rate was 89.6% in SGLT2i users, compared to 72.2% and 63.7%, respectively, in non-users. This survival benefit was observed across various cancer types, including pancreatic, hepatocellular, esophageal, head and neck, gastric, lung, colorectal, gynecologic, breast, and prostate cancers. Importantly, the inclusion criteria for the study required the presence of cancer without metastasis, and all patients had received curative treatments. However, the specific types of treatments were not disclosed in the study [86]. Moreover, a significant reduction in all-cause mortality associated with SGLT2i use was observed in heart failure patients who were cancer survivors, following a 5-year follow-up period after receiving anthracycline-based chemotherapy [74]. A similar result was reported in a cohort of colorectal cancer patients with diabetes, also over a 5-year observation period [87]. Additionally, a lower mortality risk was observed in patients with hepatocellular carcinoma who used SGLT2i during a follow-up period of 1.7 years [88]. Luo et al. demonstrated that SGLT2i use was associated with significantly improved survival outcomes in patients with non-small cell lung cancer and pre-existing diabetes, resulting in a 32% reduction in mortality risk [89]. Furthermore, lower all-cause mortality was associated with SGLT2i use during hormone therapy in prostate cancer patients [47,48].

These findings underscore the potential of SGLT2 inhibitors in combination with standard anticancer therapies to improve survival outcomes in cancer patients. While the exact mechanism behind the observed reduction in mortality remains uncertain—whether it stems from a decrease in cardiovascular risk or a possible enhancement of the anti-tumor response—ongoing clinical trials and studies are expected to provide clarity on the underlying factors driving this benefit.

## 11. Influence of SGLT2 Inhibitors on Heart Failure Incidence and Hospitalizations

While some studies have indicated a lower risk of heart failure incidence associated with SGLT2 inhibitors, the findings in certain studies did not reach statistical significance. This lack of significance may be attributed to factors such as heterogeneity in study populations or the relatively small number of patients involved [64,70]. Nonetheless, several studies consistently support the potential of SGLT2i to reduce HF hospitalization rates [44,64,65,66,67,68,86].

For instance, a study by Avula et al. [68] demonstrated that, alongside guideline-directed medical therapy, SGLT2i significantly reduced hospitalizations, HF admissions, HF incidence, and exacerbations. However, due to its retrospective design using electronic health record databases, the study did not provide data on changes in the LVEF or biomarkers.

Although Abdel-Qadir et al. reported no significant difference in the incidence of new HF [65], larger studies with greater patient populations have consistently shown a lower HF incidence among SGLT2i users [64,66,70].

In a meta-analysis by Agarwal et al., SGLT2i use was associated with a significantly lower risk of HF hospitalization and significant arrhythmias [84]. This result was further confirmed in a broader meta-analysis of nine observational studies, including various cancer types, conducted by Kuo et al. [85].

Many authors have emphasized the importance of evaluating biomarkers for the earlier identification of cardiac injury. However, due to the retrospective nature of studies, it was difficult to establish a clear role for biomarkers in the context of SGLT2i usage. Future research should incorporate biomarker-based monitoring to enhance their clinical relevance.

In summary, a comprehensive review of recent studies, including meta-analyses, has shown that SGLT2i use among oncological patients and cancer survivors is associated with a 50% reduction in HF-related hospitalizations and more than a two-thirds reduction in HF incidence. These findings suggest that SGLT2 inhibitors may play a crucial role in preventing heart failure complications in cancer patients, improving their cardiovascular health, and ultimately enhancing overall treatment outcomes [76].

Studies assessing the effectiveness of SGLT2 inhibitors in preventing cardiotoxicity following cancer treatment are consolidated in Table 1. 

## 12. Antiarrhythmic Properties of SGLT2 Inhibitors

In addition to their cardiovascular, metabolic, and renal benefits, SGLT2 inhibitors have also been associated with antiarrhythmic properties, as highlighted by several clinical studies. For instance, Gongora et al. reported a reduction in clinically significant arrhythmias among SGLT2i users [64]. Similarly, Fath et al. observed lower rates of new-onset atrial fibrillation (AF), atrial flutter, and ventricular arrhythmias in patients treated with SGLT2i [70]. These results align with those reported by Avula et al., who also noted a reduction in AF burden during various cancer treatments, further supporting the antiarrhythmic potential of SGLT2i [68]. Additionally, Koutroumpakis et al. reported a similar reduction in AF incidence in prostate cancer patients undergoing hormone therapy [47]. Perelman et al. also observed a lower incidence of AF during immunotherapy, although the study had a limited sample size, which may limit the generalizability of the findings [44]. These findings suggest that SGLT2i use may reduce the incidence of clinically significant arrhythmias in various patient populations, including those undergoing cancer treatments.

## 13. SGLT2 Inhibitors as a Remedy for Anthracycline-Induced Cardiomyopathy?

Recent pooled analyses have highlighted the potential of sodium–glucose cotransporter-2 inhibitors to confer substantial cardiovascular benefits, particularly in breast cancer patients undergoing anthracycline-based chemotherapy. In studies where more than 50% of participants received anthracyclines, a striking 99% reduction in the risk of heart failure-related hospitalizations was observed, in contrast to studies with a lower proportion of anthracycline-treated patients [76].

Ongoing and recruiting clinical trials, such as EMPACT (NCT05271162), ProtectAA (NCT06304857), PROTECT (NCT06341842), DAPA-CHEMOCARE (NCT06888505), and Cardiotoxicity in Breast Cancer Patients (NCT06491680), are set to provide valuable data on the effects of SGLT2i during chemotherapy on CTR-CVT. The outcomes of these studies will be instrumental in elucidating whether SGLT2 inhibitors can be a viable therapeutic option for mitigating cardiotoxicity in cancer treatment. Despite promising preliminary findings, the lack of evidence from randomized controlled trials has precluded the inclusion of SGLT2 inhibitors in the 2022 European Society of Cardiology (ESC) Cardio-Oncology Guidelines [4]. This highlights the critical need for RCTs to definitively determine the efficacy and safety of SGLT2 inhibitors as cardioprotective agents in cancer patients.

Preclinical and clinical evidence increasingly suggests that SGLT2 inhibitors may offer cardioprotective benefits in the context of chemotherapy-induced cardiotoxicity. However, the cardiotoxic profiles of oncological therapies are highly heterogeneous, with a significant variation in mechanisms and severity across agents such as anthracyclines, immune checkpoint inhibitors, and targeted therapies. In routine clinical practice, patients frequently undergo multimodal cancer treatments, including combinations of chemotherapy, immunotherapy, and radiotherapy. This therapeutic overlap presents a major challenge in discerning the independent cardioprotective effects of SGLT2 inhibitors. The potential confounding influence of these concurrent therapies must be carefully accounted for in any evaluation of SGLT2i efficacy.

An important direction for future research involves evaluating the therapeutic potential of SGLT2 inhibitors in non-diabetic patients undergoing cancer therapy. To date, the majority of available data originate from cohorts with established diabetes, limiting the generalizability of findings to broader oncology populations. The absence of prospective controlled studies in non-diabetic cancer patients represents a significant gap in the literature. Current cardio-oncology guidelines for the prevention of anthracycline-induced cardiomyopathy advocate for the use of beta-blockers, angiotensin-converting enzyme inhibitors (ACE inhibitors), and angiotensin receptor blockers (ARBs), which have demonstrated efficacy in preserving the LVEF during chemotherapy. However, the emerging data indicate that SGLT2i may confer additional cardioprotective benefits through mechanisms distinct from those of conventional therapies. These include an enhancement of myocardial energy efficiency, attenuation of oxidative stress, and suppression of pro-inflammatory signaling—pathways that are particularly relevant in the setting of cancer-related cardiac injury. These mechanisms may position SGLT2 inhibitors as an essential adjunct to traditional cardio-oncology strategies, complementing existing treatments for preventing heart failure during cancer therapy. Future research should also focus on elucidating the underlying molecular and physiological mechanisms by which SGLT2 inhibitors may influence tumor biology and improve overall outcomes in patients with malignancies.

Given their dual metabolic and cardioprotective properties, SGLT2 inhibitors offer a particularly compelling therapeutic approach for oncology patients presenting with multiple comorbidities. Their efficacy is especially pertinent in the setting of shared pathophysiological mechanisms such as hyperglycemia, systemic inflammation, and oxidative stress, which are prevalent both in cancer and cardiovascular disease.

As the clinical use of SGLT2 inhibitors continues to expand, particularly among patients with heart failure and chronic kidney disease, emerging data from larger multinational cohorts are expected to provide deeper insights into their potential applicability within oncological settings. To date, the safety profile of SGLT2 inhibitors in cancer patients has been favorable, with accumulating evidence supporting their tolerability and potential utility in this complex patient population.

In summary, the multifaceted therapeutic potential of SGLT2 inhibitors—including the prevention of chemotherapy-related cardiotoxicity, restoration of cardiac function in heart failure, and possible anticancer properties—positions these agents as promising candidates within the evolving field of cardio-oncology. Nonetheless, their integration into routine clinical practice will require confirmation through large-scale multicenter randomized controlled trials to establish their efficacy, safety, and applicability across diverse oncological populations.

## 14. Challenges

Long-term cardiovascular monitoring remains critical for evaluating the persistent effects of anticancer therapies, particularly anthracyclines, on cardiac function. As anthracycline-induced cardiotoxicity can present as both acute and late-onset cardiac dysfunction, ongoing randomized controlled trials are expected to provide pivotal data on the efficacy and safety of SGLT2i in mitigating CTR-CVT. Notably, growing interest surrounds the potential of SGLT2i to manage cardiotoxicity previously deemed irreversible, such as that induced by anthracyclines. However, persistent challenges—including maintaining long-term patient adherence and ensuring adequate follow-up in oncological populations—limit the comprehensive assessment of delayed cardiovascular outcomes. While preclinical models have significantly advanced our understanding of the pathophysiological mechanisms underlying anthracycline cardiotoxicity, these models often fail to fully replicate the complexity of human cardiac responses, thereby requiring cautious interpretation when extrapolating findings to clinical settings. Cardiotoxicity is a multifactorial process shaped by patient-specific variables, genetic susceptibility, and the interplay of multiple therapeutic modalities. Disentangling the specific contribution of anthracyclines from other co-administered treatments remains a formidable challenge, particularly in patients undergoing combination regimens. In light of rapid advancements in oncology, including the widespread use of targeted agents and immunotherapies, understanding how these evolving modalities interact with anthracyclines to influence cardiovascular risk is essential. A multidisciplinary precision-medicine approach will be key to optimizing cancer treatment strategies while minimizing long-term cardiovascular harm.

### Current Gaps, Problems, and Future Directions

Despite the growing interest in the use of SGLT2i in cardio-oncology, several key knowledge gaps remain, warranting further investigation. One of the primary challenges lies in the identification of patient populations most likely to benefit from SGLT2i therapy. Improved patient selection and risk stratification models will be essential to optimize the clinical application of SGLT2i in this context.

Another significant area of uncertainty is the potential for sex-based differences in the response to SGLT2i. Further studies are needed to explore whether male and female cancer patients exhibit differential responses to SGLT2i treatment, particularly with regard to cardiovascular outcomes. Additionally, a cancer type or anticancer treatment that is more prone to respond better to SGLT2i treatment should be established.

The timing of SGLT2i initiation is also a critical issue. Similarly, recommendations regarding the duration of SGLT2i therapy and criteria for discontinuation remain undefined.

Although evidence supports the cardiovascular benefits of SGLT2i as a class, there remains a lack of direct head-to-head comparisons between individual SGLT2i agents in oncology patients. Given that different SGLT2 inhibitors may have distinct pharmacodynamic and pharmacokinetic profiles, it is essential to conduct comparative effectiveness studies to better understand the relative safety and efficacy of these agents in cancer patients undergoing various forms of cancer treatment.

Addressing these gaps through targeted research, including well-designed randomized controlled trials and large-scale observational studies, will be crucial for advancing the integration of SGLT2i into clinical practice and refining their role in cardio-oncology.

Diabetes is a known risk factor for CTRCD, prompting investigation into anti-diabetic medications for cardioprotection in cancer patients. While SGLT2 inhibitors are already being explored, growing interest is emerging around GLP-1 receptor agonists (GLP-1 RAs) due to their demonstrated cardiovascular benefits. In the STEP-HFpEF trial, semaglutide improved outcomes in patients with heart failure with preserved ejection fraction (HFpEF), leading to greater improvements in exercise capacity compared to the placebo [90].

Preclinical studies suggest that GLP-1 RAs reduce pro-inflammatory markers (e.g., caspase-3, IL-1, IL-6, and TNF-α), LDL cholesterol, and senescence-related proteins while enhancing autophagic activity [91]. In a retrospective study by Chiang et al., involving 7651 cancer patients with T2DM receiving immune checkpoint inhibitors, the use of GLP-1 RAs was associated with a reduced risk of MACE (a composite of myocardial infarction, coronary revascularization, heart failure, ischemic stroke, and cardiac arrest) as well as lower all-cause mortality, without an increased risk of serious adverse events [92]. Glucagon-like peptide-1 receptor agonists (GLP-1 RAs) have demonstrated not only anti-obesity effects but also emerging potential as agents with direct anticancer activity [93]. In oncology, particularly among patients receiving chemotherapy or hormone-based treatments, managing cardiometabolic health is essential and includes addressing hyperglycemia, visceral adiposity, and dyslipidemia. GLP-1 RAs contribute to this management strategy by supporting weight reduction and maintaining stable glucose levels with minimal risk of hypoglycemia—an important factor in preserving physical strength and overall resilience during cancer therapy [93]. Moreover, early preclinical findings suggest that GLP-1 RAs may exhibit anticancer properties, interfering with tumor cell growth and inflammatory signaling in vitro, possibly through the modulation of the AMPK/mTOR and NLRP3 pathways [94].

## 15. Conclusions

Sodium–glucose cotransporter-2 inhibitors, originally developed for the management of diabetes, have emerged as promising therapeutic agents with broad cardiovascular benefits. These medications exert pleiotropic effects that extend well beyond glycemic control, offering significant advantages in heart failure management, renal function preservation, and mortality reduction. As such, SGLT2i represent a novel avenue for the prevention and management of cardiotoxicity, a growing concern in cardio-oncology.

In observational studies involving cancer patients, SGLT2 inhibitors have been associated with cardiovascular benefits and a favorable safety profile. Notably, there has been no observed increase in the risk of diabetic ketoacidosis or sepsis among patients using SGLT2i. Furthermore, emerging evidence suggests that SGLT2i, when added to guideline-directed medical therapy, may improve overall survival across various malignancies. These findings underscore the potential of SGLT2i as a promising therapeutic class that deserves further exploration in prospective clinical trials.

As research into the mechanisms of action of SGLT2 inhibitors continues to evolve, these agents may offer a valuable strategy for mitigating cardiotoxicity in cancer patients, particularly those at high risk for developing cardiovascular complications during cancer therapy. However, additional studies are needed to explore their role in cardio-oncology treatment protocols, establish optimal therapeutic strategies, and assess their long-term safety and efficacy in cancer patients.

## Figures and Tables

**Table 1:** Studies evaluating the efficacy of SGLT2i in the prevention of cardiotoxicity after oncological treatment.

First Author, Year, Ref., Country	Study Population	Tumor Type	Treatment	Mortality in SGLT2i Group vs. Non-SGLT2i	Heart Failure Hospitalizations in SGLT2i Group vs. Non-SGLT2i	Other Outcomes	Follow-Up	SGLT2i Used
**Retrospective Studies**								
Gongora et al., 2022 [64], USA	128 (32 diabetic patients on SGLT2i vs. 96 matched controls)	Various, including lymphoma, breast, genitourinary, and others	AC (DOX) and others	Lower	Lower	Reduced HF incidence and exacerbation, development of cardiomyopathy or arrhythmia, sepsis, and neutropenic fever	1.5 years	Empa, Cana, and Dapa
Abdel-Qadir et al., 2023 [65], Canada	933 (99 diabetic patients on SGLT2i vs. 843 non-SGLT2i controls)	Various (lymphoma, gastrointestinal, breast, and others)	AC (DOX and epirubicin)	No difference	Lower	Reduced HF incidence	1.6 years	Dapa, Empa, and Cana
Chiang et al., 2023 [66], Taiwan	8640 (848 diabetic SGLT patients vs. 878 non-SGLT2i controls)	Various, including gastrointestinal, genitourinary, thoracic, and others	AC, alkylating agents, antimetabolites, platinum, and plant alkaloids	Lower	Lower	Reduced HF incidence	18.8 months	Dapa, Empa, and Cana
Hwang et al., 2023 [67], Republic of Korea	81,527 (779 diabetic patients on SGLT2i vs. 77,337 non-DM controls and 3455 T2DM non-SGLT2i controls)	Various, including lymphoma, breast, genitourinary, and others	AC, HER2 inhibitors, alkylating agents, and VEGF-targeting agents	Lower	Lower	Reduced composite of HF hospitalization, acute myocardial infarction, ischemic stroke, and death	Not specified	Not specified
Avula et al., 2024 [68], Global	1280 (640 diabetic on SGLT2i vs. 640 non-SGLT2i diabetic)	Various, including lymphoma, gastrointestinal, breast, and others	AC (DOX, idarubicin, liposomal DOX, and daunorubicin), alkylating agents, antimetabolites, small-molecule tyrosine kinase inhibitors, proteasome inhibitors, radiotherapy, and others	Lower	Lower rate of heart failure admissions	Reduced hospitalizations, HF incidence and exacerbation, AF burden, AKI, and renal replacement therapy	2 years	Dapa, Empa, and Cana
Bhatti et al.; 2024 [69], USA	8675 patients on SGLT2 vs. 8675 controls	Various, including gastrointestinal, and others	AC, monoclonal Ab, proteasome inhibitors, antimetabolites, alkylating agents, small-molecule tyrosine kinase inhibitors, and others	Lower	N/A	Lower rate of CTRCD in diabetic patients treated with SGLT2i, lower rate of HF exacerbations, all-cause hospitalization, AF, and new-onset AFl	12 months	Empa, Dapa, and Cana
Fath, 2024 [70], USA	1,412 (706 patients on SGLT2i (91% diabetic) vs. 706 controls)	Various (breast, lymphoma, gastrointestinal, genitourinary, mesothelial tissue, and soft tissue)	AC (DOX, epirubicin, idarubicin, and alrubicin) and mitoxantrone	No reduction	N/A	Reduced new-onset HF, HF exacerbation, and arrhythmia	2 years	Empa, Cana, Dapa, and Ertu
Perelman et al., 2024 [44], Israel	119 (24 vs. 95)	Various, including breast, melanoma, lung, hepatoma, and others	ICI	Lower	N/A	No significant differences in MACE; we observed 0 cases of myocarditis and AF in the SGLT2i compared to 2 and 6 cases in the non-SGLT2i group, respectively	28 months	Empa and Dapa
Koutroumpakis et al., 2024 [47], USA	26,848 (2155 vs. 2155)	Prostate cancer	Hormone therapy	Lower	N/A	Lower odds of new-onset HF, HF exacerbation, PAD, AF, cardiac arrest, need for renal replacement therapy, and overall emergency room visits/hospitalizations	2 years	Cana, Dapa, and Empa
Tang, 2024 [48], USA	4312 (452 vs. 452)	Prostate cancer	Hormone therapy (GnRH agonist)	Lower	N/A	Lower incidence of HF and MI	2 years	Not specified
Henson et al., 2024 [74], USA	(1323 vs. 1323)	Various	AC	Lower	N/A	Improved survival, cachexia, and malnutrition in HF cancer survivors	5 years	Not specified
Huang et al., 2024 [86], Taiwan	50,133 (16,711 vs. 33,422)	Various (lymphoma, breast, genitourinary, and others)	AC, alkylating agents, antimicrobial agents, HER2 inhibitors, and VEGF-targeting agents	Lower	Reduction	Reduced all-cause mortality, cancer mortality, MI, and ischemic stroke	4.5 years	Not specified
Chiang et al., 2024 [87], Taiwan	1347 (92 vs. 92)	Colorectal adenocarcinoma	Monoclonal antibody, tegafur/uracil, and others	Lower	N/A	Reduced all-cause mortality	5 years	Empa, Dapa, Cana, and Ertu
Hendryx et al., 2022 [88], USA	274 (137 vs. 137)	Hepatocellular carcinoma	Surgery, chemotherapy, and radiation	Lower	N/A	Reduced all-cause mortality	1.7 years	Cana, Dapa, Empa, and Ertugliflozin
Luo et al., 2023 [89], USA	24,915 (531 on SGLT2i vs. 24,384)	Non-small cell lung cancer	Various (chemotherapy, radiation, and immunotherapy)	Lower	N/A	Improved overall survival	1.5 years	Cana, Dapa, and Empa
**Prospective Case–Control Study**								
Daniele et al., 2024 [73], EMPACARD RCT	38 vs. 38 placebo	Breast cancer	AC	No difference	No difference	Reduction in CTRCD (reduced decline in LVEF and reduced GLS impairment)	6 months	Empa 10 mg

AC: anthracyclines, AF: atrial fibrillation, AFl: atrial flutter, Cana: canagliflozin, CTRCD: cancer therapy-related cardiac dysfunction, Dapa: dapagliflozin, DOX: doxorubicin, Empa: empagliflozin, Ertu: ertugliflozin, GLS: global longitudinal strain, GnRH: gonadotropin-releasing hormone, HER2: human epidermal growth factor receptor 2, HF: heart failure, ICI: immune checkpoint inhibitor, N/A: Not Available, LVEF: left ventricular ejection fraction, MI: myocardial infarction, PAD: peripheral artery disease, RCT: randomized controlled trial, and VEGF: vascular endothelial growth factor.

## Data Availability

No new data were created or analyzed in this study. Data sharing is not applicable to this article.
